# Global Access to Uncontaminated Omega-3 Polyunsaturated Fatty Acids Requires Attention

**DOI:** 10.1016/j.focus.2025.100341

**Published:** 2025-03-28

**Authors:** Timothy H. Ciesielski

**Affiliations:** Department of Population and Quantitative Health Sciences, Case Western Reserve University School of Health Sciences, Cleveland, Ohio

## Abstract

**Figure:**
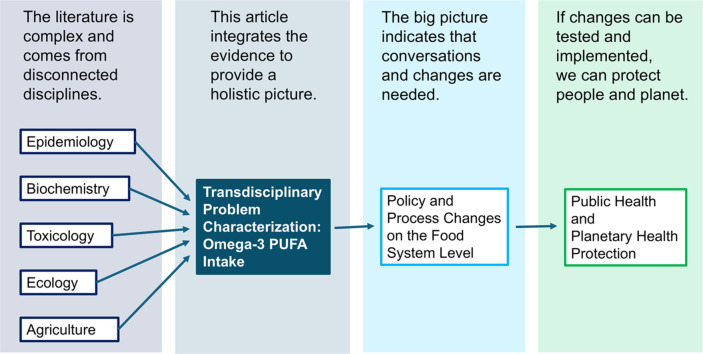

## EVIDENCE OF HUMAN HEALTH IMPACTS

The importance of Omega-3 Polyunsaturated Fatty Acids (Omega-3-PUFA) in human health has been difficult to evaluate. Various studies have evaluated multiple health outcomes, and the results have been inconsistent. However, in recent years, a series of meta-analyses have filtered through the heterogeneity to highlight stable overall patterns. These ensemble conclusions still require more scrutiny, but at present they indicate that Omega-3-PUFA intake is critical in the prevention of multiple adverse health consequences. Specifically, we see this pattern in the literature for preterm birth,^1^, ^2^, ^3^, ^4^ neurocognitive decline and dementia,^5^^,^^6^ depression and anxiety,^7^, ^8^, ^9^, ^10^, ^11^ cardiovascular disease,^12^^,^^13^ wheezing,^14^ premenstrual symptom severity,^15^ rheumatoid arthritis,^16^ several cancers (breast/prostate/colon),^17^ non-alcoholic fatty liver disease,^18^ and inflammatory biomarkers.^19^ The emergence of consensus upon meta-analysis is compelling, but the heterogeneity in the individual studies indicates that unaddressed factors are present. Most existing studies have failed to comprehensively account for the 7 factors listed in Table 1.^2^^,^^3^^,^^20^, ^21^, ^22^, ^23^, ^24^, ^25^, ^26^, ^27^, ^28^, ^29^, ^30^, ^31^, ^32^, ^33^, ^34^, ^35^, ^36^, ^37^, ^38^, ^39^ These factors add to the more commonly recognized epidemiologic pitfalls (e.g., selection bias, publication bias, and incomparable study designs), which also contribute to the heterogeneity in these findings. We should expect heterogenous findings in individual studies until researchers do a better job of addressing these factors. Additional studies, reviews, and meta-analyses are unlikely to advance our understanding if they ignore these factors. Overall, the addition of trials and mechanistic studies that characterize intermediate biomarkers offers the opportunity for strong corroboration. Although I lack the space in this editorial to fully discuss this approach across all outcomes, an example of this strategy can be found in non-alcoholic fatty liver disease.^40^ All the diseases listed above are thought to involve chronic inflammation, and if high-quality Omega-3-PUFA trials show consistent positive changes in disease-specific markers and inflammatory markers, this can provide convergent mechanistic evidence for downstream human health impacts.

**Table 1:** Key Factors Often Unaddressed: Leading to Heterogeneity in the Findings

Factor		Explanation
1	Comprehensive specification of lipids	Although it is not always easy to separate and definitively identify exact lipid contents, researchers should track and document the distinct PUFAs in their studies, to the extent possible. If supplements in a trial contain Omega-6-PUFA in addition to Omega-3-PUFA, this intrinsic confounding cannot be ignored. Extensive discussion of this topic with citations can be found in Table S1 and Table S2 from^3^.
2	Endogenous interconversion of ingested lipids^2^^,^^3^	ALA can be converted into EPA and DHA. Thus, if ALA intake is ignored, this can generate unrecognized differences in internal EPA and DHA levels. Some claim that the rate of conversion is low enough to ignore, but we know that the rate of conversion is altered by sex,^20^^,^^21^ as well as nutritional and genetic factors,^22^, ^23^, ^24^, ^25^ and some diets contain very little EPA and DHA.^26^^,^^27^ Thus, for some people, the endogenous conversion of ALA may produce a large percentage of their EPA and DHA.
3	Biochemical interplay between Omega-3 and Omega-6-PUFA^2^^,^^3^	Omega-3 and Omega-6-PUFA^2^^,^^3^ share endogenous conversion enzymes.^28^ The generally pro-inflammatory Omega-6-PUFA may process faster when Omega-3-PUFA are deficient, and the generally anti-inflammatory Omega-3-PUFA may convert slower when Omega-6-PUFA are in excess. In fact, competitive inhibition by Omega-6-PUFA such as Linoleic Acid (LA)^29^ might explain why ALA to EPA/DHA conversion rates appear to be so low. Modern humans are swimming in Omega-6 substrate.^30^ Thus, Omega-3 and Omega-6 may sometimes need to be considered together.
4	Nonlinearity in dose response	Evidence of sufficiency thresholds exists for several outcomes, and thus linear regression is often not appropriate.^2^^,^^3^ Most studies have ignored the possibility of non-linear relationships, but free-knot penalized splines^31^^,^^32^ are available and they can be used to identify putative thresholds.
5	Habitual intake vs recent intake.^2^^,^^3^	What would be the expected use of increasing intake among those who are already replete? What if they are cryptically replete in EPA and DHA because of the years of high ALA intake and endogenous conversion in the presence of low Omega-6 intake? Researchers may need blood-based biomarkers or years of dietary intake data to identify individuals who are replete.
6	Contamination of Omega-3-PUFA source foods.	Our best sources of Omega-3-PUFA are marine oils, and these are frequently contaminated with polychlorinated biphenyls (PCBs), dioxins, methylmercury, pesticides, Per- and polyfluoroalkyl substances, and plastics.^33^^,^^34^ Certainly this is an epidemiologic problem (confounding), but in a much more general sense, it is an environmental health tragedy. We have poisoned our oceans and our food. How do we eat fish in a manner that balances the intake of poisons with the intake of comingled nutrients?^35^, ^36^, ^37^ Clearly the solution is to have marine food that is not poisoned, but this would require effective pollution control. That goal is so far out of reach for the public health community that researchers simply study how to best cope with the pollution. In the near term, nutritional researchers should consider these contaminants in their work. Contaminants should either be measured and evaluated as potential confounders or the lack of this information should be listed a study limitation.
7	Lipid oxidation.^38^^,^^39^	EPA and DHA can degrade with processing or storage. This can transform lipid nutrients into lipid toxicants. In short, oxidized EPA and DHA is a problem. It is a potential confounder that needs to be considered in epidemiologic studies, and in a more general sense, it is a production problem that requires food industry and regulatory attention.

As the technical hurdles in nutritional epidemiology are addressed, the importance of Omega-3-PUFA for human health becomes clearer. It appears that we need them, and this situation has led us to wonder if we have enough of them. What follows is not a conflation of epidemiologic hurdles and supply issues, but rather a story about how hard-fought epidemiologic research consensus has led to the recognition of a global-scale supply problem.

## COUNTRY-LEVEL ANALYSES REVEAL CURRENT FOOD SYSTEM INSUFFICIENCIES

**Figure 1:**
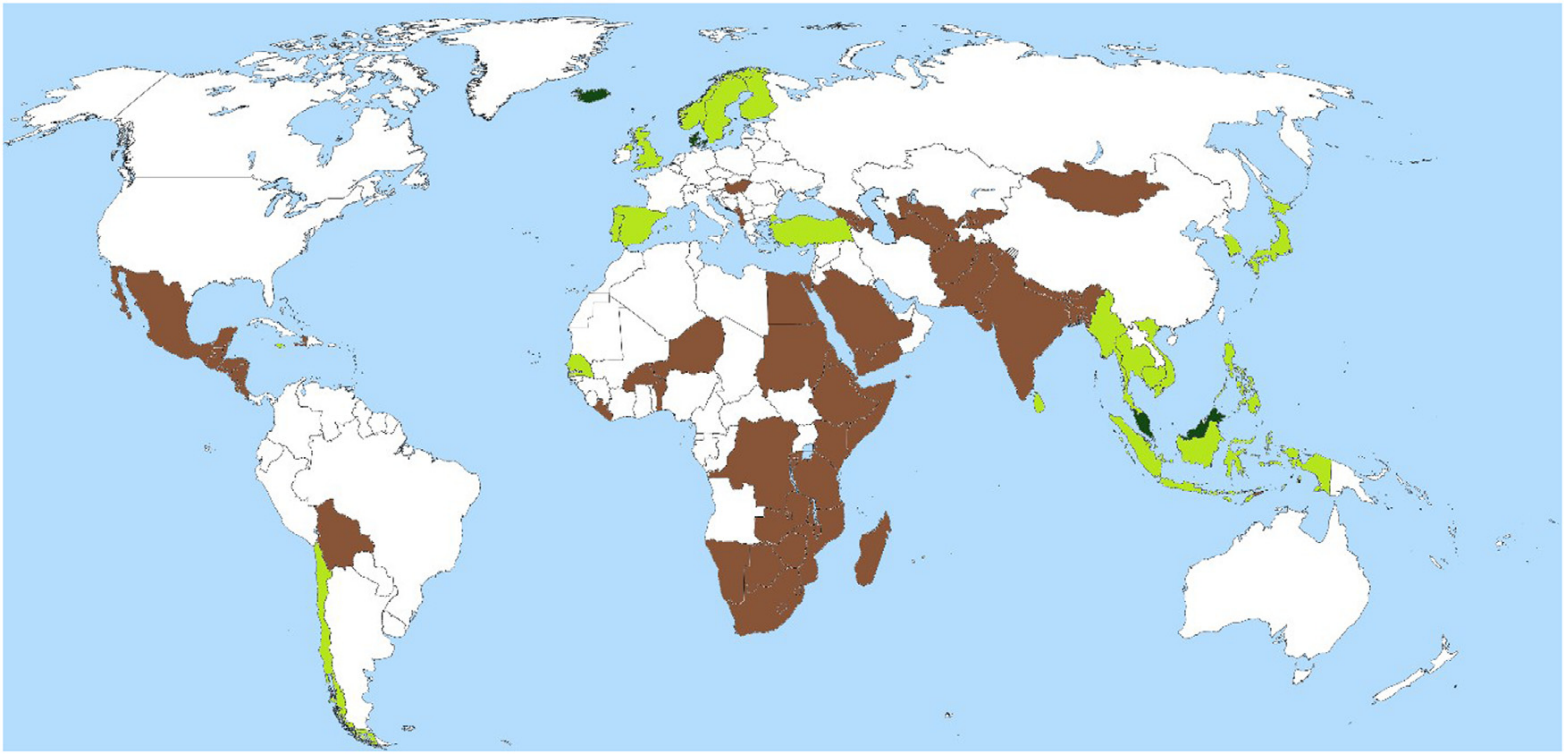
**Mean Omega-3 intakes by country - among adults.** Countries in dark green have Omega-3 intakes that are above the putative sufficiency threshold for depression (>1000 mg/day). Countries in light green have LC Omega-3-PUFA intakes that are above the putative sufficiency threshold for preterm birth (>550 mg/day). All other countries are below both thresholds (<550 mg/day). Countries in brown (n=53) have LC Omega-3-PUFA intakes <170 mg/day, and this is at least one standard deviation (380 mg/day) below the preterm birth sufficiency threshold (550 mg/day). Figure from ^4^ (re-use under the CC BY 4.0 license https://creativecommons.org/licenses/by/4.0/).

Given the sheer diversity of unaddressed factors and methodological hurdles described in Table 1,^2^^,^^3^^,^^20^, ^21^, ^22^, ^23^, ^24^, ^25^, ^26^, ^27^, ^28^, ^29^, ^30^, ^31^, ^32^, ^33^, ^34^, ^35^, ^36^, ^37^, ^38^, ^39^ we should reevaluate the scope^41^ of our global Omega-3-PUFA problems with these things in mind. National mean lipid intake estimates^26^^,^^27^ now allow us to conduct country-level analyses that account for most of these factors.^4^ The work is ongoing, but these analyses have already revealed that Omega-3-PUFA intake is insufficient in most of the world’s countries. More specifically, low intake of long-chain Omega-3-PUFA (eicosapentaenoic acid [EPA] and docosahexaenoic acid [DHA]) and their precursors (e.g., alpha-linolenic acid [ALA]) may be elevating depression and preterm birth rates in 85% of the 184 countries studied (Figure 1). Note that the unit of analysis here is the country and the inference is also on the country level. Thus, there is no cross-level inference, and the ecological fallacy is not in play. Like all analytic approaches, this method has its drawbacks, but the extensive variation in Omega-3-PUFA intakes between countries provides a rare opportunity to observe a wide exposure distribution.^42^

## ECOPHYSIOLOGY AND CLIMATE CHANGE: IT IS GOING TO GET WORSE

Additional outcomes need to be evaluated on a global scale, but we should not fail to reason with the evidence we already have. When considered together, the meta-analyses and country-level analyses indicate that Omega-3-PUFA intake is already insufficient in most of the world’s countries. Unfortunately, this problem is about to get worse. As the oceans warm, less Omega-3-PUFA is needed for cell membrane fluidity, and so less is produced by algae and less is present in marine life.^43^ This biophysical process, known as homeoviscous adaptation, is a direct function of water temperature alone.^43^ If we do not lessen the extent of ocean warming, we can expect to harvest much less Omega-3-PUFA from our oceans in the future. Unfortunately, homeoviscous adaptation is not the only threat to our already insufficient global Omega-3-PUFA access. Excess CO_2_ pollution from fossil fuel burning is acidifying the oceans, and this could result in the disruption or collapse of ocean food chains.^44^ This would leave even fewer fish to harvest from fisheries that are already strained for other reasons, such as overfishing.^45^^,^^46^ How will these mounting scarcities be distributed as richer countries exert their capacity to harvest fish and krill near poorer countries with less industrial extraction capacity?^45^ Will other species go extinct as we try to meet human lipid needs with excessive marine harvests?^47^ What new human health and ecotoxicological threats will we discover among the myriad unquantified anthropogenic ocean pollutants?^48^ PCBs, methylmercury, phthalates, flame retardants, perfluorinated chemicals, pesticides, plastics, or other as yet uncharacterized ocean pollutants could render the remaining marine Omega-3-PUFA inedible if we are not careful.^48^

## BIG PICTURE AND FUTURE DIRECTIONS

Where does this collection of transdisciplinary evidence leave us? First, although the specific sources of heterogeneity deserve more attention in future research, it must be acknowledged that meta-analyses now link Omega-3 insufficiency to a large number of adverse health outcomes. Future research may reveal even more adverse effects now that more sources of variation are being recognized and addressed. The scope of the health consequences is quite striking, and these findings beg the question: How is this possible? The key issue is that western industrialized food systems provide very low levels of Omega-3-PUFA and very high levels of Omega-6-PUFA. This distinction is a new development in the history of our species. We evolved in food environments where Omega-6 and Omega-3-PUFA were approximately equal in abundance.^30^ Now our industrialized diets contain about 20 times more Omega-6-PUFA than Omega-3-PUFA.^30^ This is not to say that I know exactly how to fix our food production or that it can be de-industrialized at our current population levels. It is to say that we need our food to provide more Omega-3-PUFA and less Omega-6-PUFA.

When we look at the food system level, we find evidence that mean Omega-3-PUFA intake is deficient in 85% of the countries studied to date. Contamination, overfishing, ocean acidification, and climate change will likely make clean Omega-3-PUFA even less available in the near future.^33^^,^^34^^,^^43^^,^^44^^,^^49^ Having said this, we know that ∼15% of countries have achieved sufficiency (at least with respect to preterm birth), and we might learn something from them. These countries are quite diverse, but if we look at Figure 1, we see that all of them share one feature: extensive ocean access. Thus, we must protect the oceans, steward fisheries, and only engage in aquaculture^50^ that is sustainable or regenerative. However, this still may not suffice. We might need to develop sustainable terrestrial sources of long-chain Omega-3-PUFA, such as Purslane.^51^ We might also need to consider the possibility of addition by subtraction. Reducing mean Omega-6 intakes may make it easier to achieve Omega-3 sufficiency in many settings. This will be especially true if we need to lean heavily on the endogenous conversion of ALA, because ALA is much easier to get from terrestrial plants than EPA or DHA.

Finally, we need to step back and evaluate our frames of inquiry. If 85% of earth’s countries do not have a high enough mean daily intake of this class of lipids, is this a healthcare problem or a public health problem? Are supplements a safe, equitable, scalable, and optimally efficacious solution? Is food-based access to sufficient, uncontaminated, non-oxidized Omega-3-PUFA a human right? Is this a planetary health problem? We need to collectively consider these issues soon, objectively, and in good faith.
